# Consumption of whole grains and cereal fiber and total and cause-specific mortality: prospective analysis of 367,442 individuals

**DOI:** 10.1186/s12916-015-0294-7

**Published:** 2015-03-24

**Authors:** Tao Huang, Min Xu, Albert Lee, Susan Cho, Lu Qi

**Affiliations:** Department of Nutrition, Harvard School of Public Health, 665 Huntington Ave, Boston, MA 02115 USA; NutraSource (AWL), Royal Oak, MI 48073 USA; NutraSource (SSC), Clarksville, MD 21029 USA; Channing Laboratory, Department of Medicine, Brigham and Women’s Hospital and Harvard Medical School, 75 Francis St, Boston, MA 02115 USA

**Keywords:** Cereal fiber, Mortality, Whole grains

## Abstract

**Background:**

Intakes of whole grains and cereal fiber have been inversely associated with the risk of chronic diseases; however, their relation with total and disease-specific mortality remain unclear. We aimed to prospectively assess the association of whole grains and cereal fiber intake with all causes and cause-specific mortality.

**Methods:**

The study included 367,442 participants from the prospective NIH-AARP Diet and Health Study (enrolled in 1995 and followed through 2009). Participants with cancer, heart disease, stroke, diabetes, and self-reported end-stage renal disease at baseline were excluded.

**Results:**

Over an average of 14 years of follow-up, a total of 46,067 deaths were documented. Consumption of whole grains were inversely associated with risk of all-cause mortality and death from cancer, cardiovascular disease (CVD), diabetes, respiratory disease, infections, and other causes. In multivariable models, as compared with individuals with the lowest intakes, those in the highest intake of whole grains had a 17% (95% CI, 14–19%) lower risk of all-cause mortality and 11–48% lower risk of disease-specific mortality (all *P* for trend <0.023); those in the highest intake of cereal fiber had a 19% (95% CI, 16–21%) lower risk of all-cause mortality and 15–34% lower risk of disease-specific mortality (all *P* for trend <0.005). When cereal fiber was further adjusted, the associations of whole grains with death from CVD, respiratory disease and infections became not significant; the associations with all-cause mortality and death from cancer and diabetes were attenuated but remained significant (*P* for trend <0.029).

**Conclusions:**

Consumption of whole grains and cereal fiber was inversely associated with reduced total and cause-specific mortality. Our data suggest cereal fiber is one potentially protective component.

## Background

Grains, also called cereals, are the seeds of plants cultivated for food. When whole, they include the germ, bran, and endosperm [1]. Most whole grains are abundant sources of dietary fiber and other nutrients, such as minerals and antioxidants, which have shown beneficial effects on human health including improvement of weight loss, insulin sensitivity, and lipid profile, as well as inhibition of systemic inflammation [2-4].

In epidemiology studies, evidence is accumulating indicating that consumption of whole grain products or their effective components, especially dietary fiber found in the grain, i.e., cereal fiber, may reduce the risk of chronic disease. Several recent meta-analyses taking into account a large number of subjects and prospective studies showed significant and consistent protective effects of high intake of whole grains and cereal fiber on type 2 diabetes [5], cardiovascular disease (CVD) [6], and certain cancers (e.g., colorectal cancer) [7]. In our earlier analysis in the Nurses’ Health Study, we observed potential protective effects of whole grains on total or cardiovascular mortality in diabetic women [8]. Although the National Institutes of Health (NIH)-AARP Diet and Health Study previously reported that dietary fiber intake was inversely associated with the risk of total death and death from CVD, infectious diseases, and respiratory diseases [9], few studies have prospectively examined the associations of whole grains and its components, such as cereal fiber, with total or disease-specific mortality.

In the present study, we used data from 367,442 people who were at risk for a total of 12.3 million person-years. We aimed to provide reliable estimates of independent associations between baseline whole grains and cereal fiber intake and the risk of total or cause-specific death from CVD, cancers, diabetes, and other diseases.

## Methods

### Study population

The NIH-AARP Diet and Health Study included 566,399 AARP members aged 50 to 71 from six US states (California, Florida, Louisiana, New Jersey, North Carolina, and Pennsylvania) and two metropolitan areas (Atlanta, Georgia, and Detroit, Michigan) [10]. Participants responded to a questionnaire mailed in October 1995 and December 1997. Details of the NIH-AARP Study have been previously described [11]. Among participants who returned the questionnaires with satisfactory dietary data, we excluded individuals who indicated that they were proxies for the intended respondent (n = 15,760) as well as those who had cancer (n = 50,591), heart disease (n = 80,254), stroke (n = 12,812), diabetes (n = 52,647), or self-reported end-stage renal disease at baseline (n = 1,299). We also excluded those who reported extreme consumption (>2 times the interquartile ranges of Box-Cox transformed intake) of total energy (n = 3,771) and dietary fiber (n = 3,324). Exclusion of individuals reporting extreme energy intake is widely used in nutritional epidemiology studies since these participants are more likely to over- or under-report their intake [12]. After exclusions (n = 198,957), the analytic cohort included 367,442 individuals. The NIH-AARP Diet and Health study was approved by the Special Studies Institutional Review Board of the US National Cancer Institute. All participants provided written informed consent.

### Assessment of dietary exposures

At baseline, dietary intake was assessed with a self-administered 124-item food frequency questionnaire (FFQ), which was an early version of the Diet History Questionnaire developed at the National Cancer Institute [13,14]. Participants were asked to report their usual frequency of intake and portion size over the past 12 months using 10 predefined frequency categories ranging from never to 6+ times per day for beverages and from never to 2+ times per day for solid foods with three portion size categories. The food items, portion sizes, and nutrient database were constructed using the US Department of Agriculture’s 1994–1996 Continuing Survey of Food Intakes by Individuals. The FFQ used in the study was calibrated using two non-consecutive 24-hour dietary recalls in 1953 NIH-AARP study participants. The nutrient database for dietary fiber was based on AOAC methods.

The whole grains were defined as the whole grain part of each product. The US Department of Agriculutre’s Pyramid Servings Database enabled us to accurately estimate whole-grain intake from all foods in the FFQ. The sources of whole-grain intake in the FFQ used in our study were ready-to-eat cereals, high-fiber cereals, other fiber cereals, whole-grain breads or dinner rolls, cooked cereal, popcorn, pancakes, waffles, French toast or crepes, rice or other cooked grains, bagels, English muffins, tortillas, pasta, crackers, chips, cookies or brownies, sweet pastries, and pies. In this Continuing Survey of Food Intakes by Individuals dataset., whole grain foods were defined as those containing at least 25% whole grains and/or bran. Main fibers are from fruit, grains, vegetables, and beans in the present study. Cereal fiber was defined as fiber from all cereals (e.g., ready-to-eat cereals, high-fiber cereals, cooked cereal, and other fiber cereals) and grain-based products.

In specifying numbers of deaths by intake quintile, the number of deaths is determined by the energy-adjusted intake quintile for the entire population; when deaths are specific to a sex, we used the quintiles within the sex. We also collected demographic, anthropometric, and lifestyle information, including history of smoking (the number of cigarettes smoked per day), time of smoking cessation (<1 years, 1 to 5 years, 5 to 10 years, or ≥10 years before baseline), physical activity (never, rare, 1 to 2, 3 to 4, ≥5 hours/week), alcohol intake (g/day) family history of cancers, menopausal hormone therapy use in women, and some medical conditions at baseline.

### Ascertaining mortality

The AARP dataset denotes date of death and cause of death. There are 22 broad categories for cause of death. The modeling analysis for specific cause of death is performed with the study end date of 2008. For total mortality, models for study end dates in 2008 and 2009 were designed. Subjects with date of death after the study’s end date are treated as alive at the end of the study, with no death or cause of death in the model. When the study end date was 2008, and there was a date of death but no cause of death for 2008 or earlier, the subject was not included in the modeling for cause of death, but only total mortality. Thus, when specifying numbers of deaths it depends on both the study end date and whether the cause of death field is missing. Vital status was determined through a periodic linkage of the cohort to the Social Security Administration Death Master File and follow-up searches of the National Death Index Plus for participants who matched the Social Security Administration Death Master File, cancer registry linkage, questionnaire responses, and responses to other mailings. The *International Classification of Diseases, Ninth Revision* [15] and the *International Statistical Classification of Diseases, 10th Revision* [16] were used to define death as follows: cancer (ICD-9, 140–239; ICD-10, C00–C97 and D00–D48), CVD (ICD-9, 390–398, 401–404, 410–429, and 440–448; ICD-10, I00–I13, I20–I51, and I70–I78), diabetes (ICD-9, 250; ICD-10, E10–E14), respiratory disease (ICD-9, 480–487 and 490–496; ICD-10, J10–J18 and J40–J47), infections (ICD-9, 001–139; ICD-10, A00–B99), and all other/unknown causes.

### Statistical analysis

We used the Cox proportional hazards model to estimate hazard ratios (HRs) and two-sided 95% confidence intervals (CIs) using the SAS PROC PHREG procedure (Version 9.1; SAS Institute Inc., Cary, NC, USA). Person-years of follow-up were calculated from the date of the baseline questionnaire until the date of death or the end of follow-up (December 31, 2009), whichever occurred first. Intake of whole grains and cereal fiber were adjusted for total energy intake using the residual method [17], and were categorized into quintiles.

We presented the results from four analysis models. Model 1, adjusted for age and gender; Model 2, adjusted for age, gender, the number of cigarettes smoked per day, and time of smoking cessation (<1 years, 1 to 5 years, 5 to 10 years, or ≥10 years before baseline); Model 3, adjusted for age, gender, the number of cigarettes smoked per day, time of smoking cessation (<1 years, 1 to 5 years, 5 to 10 years, or ≥10 years before baseline), race or ethnicity group, alcohol intake, education level, marital status (yes, no), health status (poor, fair, good, very good), obesity (underweight, overweight, obesity), physical activity, consumption of red meat (processed and fresh meat), total fruit and total vegetables, total energy intake, and hormone usage; and Model 4, based on Model 3 further adjusted for cereal fiber (whole grains analysis).

For missing data in each covariate, we created indicator variables. Overall, missing data was less than 5%. The model results summary includes the results of statistical tests for trend in the response for the risk variable. Quintiles Trend *P* denotes the *P* value when the median value within the risk variable quintile is included in the hazard model as linear.

## Results

Table 1 shows baseline characteristics of study participants (n = 367,442), according to intake of whole grains and cereal fiber. During an average of 14 years of follow-up (total person-years, 5,148,760), we documented 46,067 deaths, among them 11,283 from CVD, 19,043 from cancer, 371 from diabetes, 3,796 from respiratory disease, 922 from infection, and 5,223 from other causes. At baseline, intakes of whole grains and cereal fiber were inversely correlated with prevalence of overweight, obesity, and current smoking, as well as intake of red meat. The levels of moderate and vigorous physical activity were higher among participants with higher intakes of whole grains or cereal fiber than those with lower intakes.

**Table 1:** **Baseline characteristics of the study participants according to intake of whole grains and cereal fiber**

	**Total participants**	**Whole grains**			**Cereal fiber**		
		**Q1**	**Q3**	**Q5**	**Q1**	**Q3**	**Q5**
n	367,442	73,488	73,489	73,489	73,488	73,489	73,489
Age, mean years	61.7	61.1	61.7	62.1	61.0	61.7	62.2
Female, %	43.9	30.7	51.9	40.4	35.0	49.9	40.2
Whites, %	92.9	92.6	92.9	93.2	91.2	93.0	94.4
College graduate, %	41.0	35.2	41.4	44.9	36.2	40.4	46.4
Married, %	68.1	72.7	65.2	68.9	69.8	66.1	69.9
Moderate physical activity (3–4 times/week), %	27.3	23.0	27.8	30.6	23.6	27.2	30.9
Vigorous physical activity (≥5 times/week), %	19.2	17.5	18.0	23.6	18.1	17.6	24.1
Overweight, %	42.4	44.5	42.0	41.0	43.9	42.2	41.1
Obesity, %	19.6	22.7	19.7	16.6	23.6	20.0	15.0
Very good or excellent self-report health, %	61.4	56.5	62.2	64.3	58.8	61.4	65.0
Previous or current use of postmenopausal hormone therapy, %	55.0	47.9	55.6	58.1	45.5	54.4	59.2
Former smoker, %	48.7	48.5	48.2	49.5	47.6	48.4	50.2
Current smoker, %	12.8	21.4	11.3	8.0	21.4	11.5	7.0
Median total energy intake (kcal/d)	1,805	2,394	1,527	1,855	2,330	1,563	1,832
Median alcohol intake (g/d)	14.7	32.5	10.0	8.8	27.5	11.6	9.9
Median servings of food							
Red meat (oz/d)	2.0	3.1	1.6	1.7	3.1	1.71	1.6
Fruits (cup eq/d)	2.0	2.1	1.8	2.2	2.3	1.8	2.2
Vegetables (cup eq/d)	1.9	2.2	1.7	2.0	2.3	1.7	2.0

### Whole grains and cereal fiber intake with total mortality

In age- and gender-adjusted analysis (Model 1), we found that intake of whole grains were inversely associated with all-cause mortality (Table 2). As compared with the lowest quintile, the HRs across increasing quintiles of whole grain intake were 0.78 (95% CI, 0.76–0.80), 0.70 (95% CI, 0.68–0.72), 0.63 (95% CI, 0.61–0.65), and 0.61 (95% CI, 0.59–0.62) (*P* trend <0.0001). Further adjustment for smoking status and time since smoking cessation (Model 2) did not appreciably change the associations. When the models further included race/ethnicity, education, marital status, self-rated health status, obesity (underweight, overweight, and obesity), physical activity, use of menopausal hormone therapy, and intake of alcohol, red meat, fruits, vegetables, and total energy (Model 3), the highest quintile of whole grain intake was associated with 17% (95% CI, 14–19%) lower risk of all-cause mortality (*P* trend <0.0001). The associations between whole grain intake and all-cause mortality was attenuated, the highest quintile of whole grain intake was associated with 6% (95% CI, 3–10%) lower risk, but remained significant when cereal fiber was additionally adjusted (Model 4; *P* trend = 0.002). These results suggested that the protective effects of whole grain may be due, at least in the main part, to its cereal fiber component.

**Table 2:** **Association of whole grain intake with total and cause-specific mortality**

	**All participants**	**Whole grains (oz eq/d)**					***P*** **trend**
		**Q1 (n = 41,248)**	**Q2 (n = 41,248)**	**Q3 (n = 41,249)**	**Q4 (n = 41,248)**	**Q5 (n = 41,249)**	
		**0.13**	**0.30**	**0.47**	**0.69**	**1.20**	
**Causes of death**							
**All cause**							
No. of deaths	46,067	11,845	9,450	8,694	8,054	8,024	
Model 1		1.00	0.78 (0.76–0.80)	0.70 (0.68–0.72)	0.63(0.61–0.65)	0.61 (0.59–0.62)	<0.0001
Model 2		1.00	0.88 (0.86–0.91)	0.83 (0.81–0.85)	0.78 (0.75–0.80)	0.77 (0.75–0.79)	<0.0001
Model 3		1.00	0.93 (0.90–0.95)	0.89 (0.87–0.92)	0.85 (0.82–0.87)	0.83 (0.81–0.86)	<0.0001
Model 4		1.00	0.96 (0.93–0.99)	0.95 (0.92–0.98)	0.92 (0.89–0.96)	0.94 (0.90–0.97)	0.002
**Cardiovascular disease**							
No. of deaths	11,283	2,921	2,330	2,121	1,914	1,997	<0.0001
Model 1		1.00	0.78 (0.74–0.83)	0.69 (0.65–0.73)	0.60 (0.57–0.64)	0.60 (0.57–0.64)	<0.0001
Model 2		1.00	0.88 (0.83–0.93)	0.81 (0.77–0.86)	0.73 (0.69–0.77)	0.75 (0.71–0.80)	<0.0001
Model 3		1.00	0.93 (0.88–0.98)	0.88 (0.83–0.93)	0.81 (0.77–0.86)	0.83 (0.78–0.88)	<0.0001
Model 4		1.00	0.96 (0.91–1.02)	0.95 (0.89–1.01)	0.90 (0.84–0.97)	0.95 (0.88–1.03)	0.188
**Cancer**							
No. of deaths	19,043	4,836	3,912	3,616	3,388	3,291	
Model 1		1.00	0.79 (0.76–0.83)	0.71 (0.68–0.75)	0.65 (0.62–0.68)	0.61 (0.59–0.64)	<0.0001
Model 2		1.00	0.91 (0.87–0.95)	0.86 (0.83–0.90)	0.82 (0.79–0.86)	0.80 (0.76–0.84)	<0.0001
Model 3		1.00	0.94 (0.90–0.98)	0.91 (0.87–0.95)	0.88 (0.84–0.92)	0.85 (0.81–0.89)	<0.0001
Model 4		1.00	0.96 (0.92–1.00)	0.95 (0.90–0.99)	0.93 (0.88–0.98)	0.93 (0.88–0.99)	0.025
**Diabetes**							
No. of deaths	371	113	72	73	66	47	
Model 1		1.00	0.62 (0.46–0.84)	0.62 (0.46–0.83)	0.54 (0.40–0.73)	0.37 (0.27–0.52)	<0.0001
Model 2		1.00	0.67 (0.49–0.90)	0.67 (0.50–0.91)	0.60 (0.44–0.82)	0.42 (0.30–0.60)	<0.0001
Model 3		1.00	0.71 (0.53–0.96)	0.76 (0.56–1.03)	0.72 (0.53–0.99)	0.52 (0.37–0.75)	0.0009
Model 4		1.00	0.74 (0.54–1.00)	0.81 (0.59–1.13)	0.78 (0.55–1.13)	0.57 (0.37–0.89)	0.029
**Respiratory disease**							
No. of deaths	3,796	1,123	802	673	606	592	
Model 1		1.00	0.69 (0.63–0.75)	0.56 (0.50–0.61)	0.48 (0.43–0.53)	0.45 (0.41–0.50)	<0.0001
Model 2		1.00	0.88 (0.80–0.96)	0.79 (0.72–0.87)	0.74 (0.67–0.82)	0.74 (0.67–0.82)	<0.0001
Model 3		1.00	0.99 (0.90–1.09)	0.94 (0.85–1.03)	0.91 (0.82–1.01)	0.89 (0.80–0.98)	0.0099
Model 4		1.00	1.02 (0.93–1.12)	1.00 (0.90–1.11)	1.01 (0.90–1.14)	1.03 (0.91–1.18)	0.67
**Infections**							
No. of deaths	922	251	184	163	161	163	
Model 1		1.00	0.71 (0.59–0.86)	0.61 (0.50–0.75)	0.58 (0.48–0.71)	0.57 (0.47–0.70)	<0.0001
Model 2		1.00	0.78 (0.64–0.94)	0.69 (0.57–0.84)	0.68 (0.55–0.83)	0.68 (0.55–0.83)	0.0002
Model 3		1.00	0.84 (0.70–1.02)	0.78 (0.64–0.96)	0.79 (0.65–0.97)	0.77 (0.62–0.95)	0.02
Model 4		1.00	0.87 (0.71–1.06)	0.83 (0.67–1.04)	0.87 (0.69–1.10)	0.89 (0.68–1.16)	0.54
**Other/unknown causes**							
No. of deaths	5,223	1,206	1,058	1,038	940	981	
Model 1		1.00	0.86 (0.79–0.93)	0.82 (0.76–0.90)	0.71 (0.66–0.78)	0.72 (0.66–0.78)	<0.0001
Model 2		1.00	0.91 (0.84–0.99)	0.90 (0.83–0.98)	0.80 (0.73–0.87)	0.81 (0.74–0.88)	<0.0001
Model 3		1.00	0.97 (0.88–1.06)	0.97 (0.89–1.06)	0.87 (0.79–0.96)	0.86 (0.78–0.94)	0.0001
Model 4		1.00	0.99 (0.91–1.09)	1.03 (0.93–1.13)	0.96 (0.86–1.06)	0.98 (0.87–1.09)	0.54

Data are hazard ratios (HR) and 95% confidence interval (CI). The numbers of deaths are for participants who died during follow-up. The co-variables are baseline assessments.

Model 1, Adjusted for age and gender; Model 2, Adjusted for age, gender, the number of cigarettes smoked per day, and time of smoking cessation (<1 years, 1 to 5 years, 5 to 10 years, or ≥10 years before baseline); Model 3, Adjusted for age, gender, the number of cigarettes smoked per day, time of smoking cessation (<1 years, 1 to 5 years, 5 to 10 years, or ≥10 years before baseline), race or ethnicity group, alcohol intake, education level, marital status (yes, no), health status (poor, fair, good, very good), obesity (underweight, overweight, obesity), physical activity, consumption of red meat, total fruit and total vegetables, total energy intake, and hormone usage. Model 4, Based on Model 3 further adjusted for cereal fiber.

Similarly, we found that cereal fiber intake was significantly associated with all-cause mortality in age- and gender-adjusted and multivariate-adjusted models (Models 1, 2 and 3; all *P* trend <0.0001; Table 3). In model 3, the highest quintile of cereal intake was associated with 19% (16–21%) lower risk of all-cause mortality (*P* trend <0.0001).

**Table 3:** **Association of cereal fiber intake with total and cause-specific mortality**

	**All participants**		**Cereal fiber (g/d)**				***P*** **trend**
		**Q1 (n = 73,488)**	**Q2 (n = 73,488)**	**Q3 (n = 73,489)**	**Q4 (n = 73,488)**	**Q5 (n = 73,489)**	
		**2.02**	**4.15**	**5.27**	**6.65**	**10.22**	
**Causes of death**							
**All cause**							
No. of deaths	46,067	11,700	9,652	8,664	8,133	7,918	
Model 1		1.00	0.80 (0.78–0.83)	0.70 (0.68–0.72)	0.64 (0.62–0.66)	0.59 (0.58–0.61)	<0.0001
Model 2		1.00	0.89 (0.87–0.92)	0.83 (0.81–0.85)	0.78 (0.76–0.81)	0.76 (0.73–0.78)	<0.0001
Model 3		1.00	0.93 (0.90–0.95)	0.87 (0.85–0.90)	0.84 (0.81–0.86)	0.81 (0.79–0.84)	<0.0001
**Cardiovascular disease**	11,283						
No. of deaths		2,901	2,368	2,094	1,986	1,934	
Model 1		1.00	0.80 (0.76–0.85)	0.69 (0.65–0.73)	0.63 (0.59–0.66)	0.57 (0.54–0.61)	<0.0001
Model 2		1.00	0.88 (0.83–0.93)	0.80 (0.76–0.85)	0.76 (0.71–0.80)	0.72 (0.68–0.76)	<0.0001
Model 3		1.00	0.93 (0.87–0.98)	0.86 (0.81–0.91)	0.83 (0.78–0.88)	0.80 (0.75–0.85)	<0.0001
**Cancer**							
No. of deaths	19,043	4,772	3,974	3,616	3,391	3,290	
Model 1		1.00	0.81 (0.78–0.85)	0.72 (0.69–0.76)	0.66 (0.63–0.69)	0.61 (0.59–0.64)	<0.0001
Model 2		1.00	0.91 (0.87–0.95)	0.87 (0.83–0.91)	0.83 (0.79–0.86)	0.80 (0.77–0.84)	<0.0001
Model 3		1.00	0.94 (0.90–0.98)	0.90 (0.86–0.95)	0.87 (0.83–0.91)	0.85 (0.81–0.89)	<0.0001
**Diabetes**							
No. of deaths	371	92	92	72	57	51	
Model 1		1.00	0.92 (0.69–1.22)	0.70 (0.52–0.95)	0.54 (0.39–0.74)	0.45 (0.32–0.64)	<0.0001
Model 2		1.00	0.97 (0.73–1.29)	0.77 (0.56–1.04)	0.60 (0.43–0.83)	0.52 (0.37–0.73)	<0.0001
Model 3		1.00	1.00 (0.74–1.35)	0.83 (0.60–1.15)	0.70 (0.50–0.99)	0.66 (0.46–0.94)	0.005
**Respiratory disease**							
No. of deaths	3,796	1,082	866	652	649	547	
Model 1		1.00	0.75 (0.68–0.82)	0.54 (0.49–0.59)	0.52 (0.47–0.57)	0.42 (0.38–0.46)	<0.0001
Model 2		1.00	0.91(0.83–1.00)	0.76 (0.69–0.84)	0.80 (0.72–0.88)	0.70 (0.63–0.78)	<0.0001
Model 3		1.00	0.98 (0.89–1.08)	0.82 (0.74–0.91)	0.89 (0.80–0.99)	0.79 (0.71–0.88)	<0.0001
**Infections**							
No. of deaths	922	230	210	165	157	160	
Model 1		1.00	0.87 (0.72–1.05)	0.66 (0.54–0.81)	0.61 (0.50–0.75)	0.60 (0.49–0.74)	<0.0001
Model 2		1.00	0.94 (0.78–1.13)	0.75 (0.61–0.91)	0.71 (0.58–0.87)	0.71 (0.58–0.88)	0.0002
Model 3		1.00	1.04 (0.85–1.27)	0.84 (0.68–1.05)	0.82 (0.66–1.02)	0.83 (0.67–1.03)	0.023
**Other/unknown causes**							
No. of deaths	5,223	1,215	1,050	1,044	963	951	
Model 1		1.00	0.84 (0.77–0.91)	0.81 (0.74–0.88)	0.72 (0.66–0.78)	0.67 (0.62–0.73)	<0.0001
Model 2		1.00	0.88 (0.81–0.96)	0.88 (0.81–0.95)	0.79 (0.73–0.87)	0.76 (0.70–0.83)	<0.0001
Model 3		1.00	0.90 (0.83–0.99)	0.91 (0.83–0.99)	0.83 (0.76–0.91)	0.79 (0.72–0.87)	<0.0001

Data are hazard ratios (HR) and 95% confidence interval (CI). The numbers of deaths are for participants who died during follow-up. The co-variables are baseline assessments.

Model 1, Adjusted for age and gender; Model 2, Adjusted for age, gender, the number of cigarettes smoked per day, and time of smoking cessation (<1 years, 1 to 5 years, 5 to 10 years, or ≥10 years before baseline); Model 3, Adjusted for age, gender, the number of cigarettes smoked per day, time of smoking cessation (<1 years, 1 to 5 years, 5 to 10 years, or ≥10 years before baseline), race or ethnicity group, alcohol intake, education level, marital status (yes, no), health status (poor, fair, good, very good), obesity (underweight, overweight, obesity), physical activity, consumption of red meat, total fruit and total vegetables, total energy intake, and hormone usage.

### Whole grains and cereal fiber intake with cause-specific mortality

We next tested the associations for cause-specific mortalities. In age- and gender-adjusted and multivariate adjusted models (Models 1, 2 and 3), intakes of whole grains or cereal fiber were inversely associated with risk of death from CVD, cancer, diabetes, respiratory disease, infections, and other/unknown causes (all *P* trend <0.023). In Model 3, as compared with the lowest quintiles, people in the highest quintile of whole grain intake had 11% (respiratory disease) to 48% (diabetes) lower risk of cause-specific mortality, while people in the highest quintile of cereal fiber intake had 15% (cancer) to 34% (diabetes) lower risk of cause-specific mortality.

When cereal fiber was further adjusted, the associations of whole grains with death from CVD, respiratory disease, infections, and other causes became non-significant; however, the associations with death from cancer and diabetes remained significant (*P* trend <0.029).

## Discussion

In this large prospective cohort study of the US population, we found that high consumption of whole grains or cereal fiber was significantly associated with reduced risk of all-cause mortality and death from CVD, cancer, diabetes, respiratory disease, infections, and other causes. As compared with individuals with the lowest intake of whole grains, those in the highest intake group had a 17% lower risk of all-cause mortality and 11 to 48% lower risk of disease-specific mortality. As compared with individuals with the lowest intake of cereal fiber, those in the highest intake group had a 19% lower risk of all-cause mortality and 15 to 34% lower risk of disease-specific mortality. Furthermore, our results suggested that the protective effects of whole grains may due, at least in the main part, to its cereal fiber component.

To the best of our knowledge, the present study is, thus far, the largest in size regarding deaths in a prospective setting. Our findings are concordant with previously observed protective effects of whole grain intake on CVD, diabetes, and certain cancers [18,19]. Based on a meta-analysis of six cohort studies including 286,125 participants and 10,944 cases, a two servings per day increment in whole grain consumption was associated with a 21% (95% CI, 13–28%) decrease in risk of type 2 diabetes after adjustment for potential confounders and BMI [5]. These findings were confirmed by Ye et al.’s meta-analysis [20], in which it was also reported that compared with never/rare consumers of whole grains, individuals consuming 48 to 80 g of whole grains per day (3 to 5 serving/day) had a 21% lower risk of CVD (relative risk = 0.79; 95% CI, 0.74–0.85). Inverse associations were also reported between intake of whole grains and incident hypertension [21]. In a meta-analysis of 25 prospective studies, the summary relative risk of developing colorectal cancer for 10 g daily of cereal fiber was 0.90 (95% CI, 0.83–0.97), pooled results from six studies showed the relative risk for an increment of three servings daily of whole grains was 0.83 (95% CI, 0.78–0.89) [7]. High whole grain intakes have been related to reduced risk of other cancers, such as digestive cancer, in prospective studies, although the protective effects were not consistently observed [22,23].

Very few previous studies have examined the relationship between whole grains and their components with mortality in humans. Our findings are consistent with the results reported in the Nurses’ Health Study, in which whole grain intake, especially bran, was associated with lower all-cause and CVD mortality in diabetic women [24]. Similarly, higher fiber intake was associated with lower total mortality, particularly mortality from circulatory, digestive, and non-CVD non-cancer inflammatory diseases in a large European prospective study of 452,717 men and women [25]. In a previous analysis among our study samples, it was found that intake of fiber from grains but not from other sources was inversely related to all-cause mortality and death from cancer, CVD, infections, and respiratory disease [9]. In this updated analysis, we found cereal fiber intake was inversely associated with death from diabetes. However, we did not report the associations of specific types of whole grain foods/products with mortality and cause-specific mortality, since it is hard to further differentiate such food groups; this presents a limitation of this observational study.

In addition, we found that the associations of whole grains with death from CVD, respiratory disease, and infections became non-significant after adjustment for cereal fiber intake. The associations with total mortality and death from cancer and diabetes were also largely attenuated, although they remained significant after adjustment for cereal fiber intake. These observations suggest that the protective effects of whole grains on mortality are at least partly mediated by its cereal fiber component. Such a postulation is supported by previous evidence that shows cereal fiber intake is related to an improvement of insulin sensitivity and lipid profile, an increase in protective molecules such as adiponectin, and a reduction in inflammation markers [26-28].

The protective effect of whole grains and fiber consumption on risk of mortality is biologically plausible. Dietary fiber intake is associated with lower levels of inflammation markers, such as C-reactive protein, and tumor necrosis factor α receptor 2, which play key roles in chronic inflammatory conditions [29,30]. Whole grain foods are rich in fiber. Therefore, the anti-inflammatory effects of dietary fiber may help explain, at least in part, the inverse associations of whole grains and fiber consumption with chronic disease death. Moreover, whole grains and cereal fiber have a high content of antioxidants, vitamins, trace minerals, phenolic acids, lignans, and phytoestrogens, which have been associated with a reduced risk of colorectal cancer [31] and lower risk of death from non-cardiovascular, non-cancer inflammatory diseases and respiratory system diseases [32]. In addition, dietary fibers have specific and unique impacts on intestinal microbiota composition and metabolism [33,34]. Additionally, recent studies have related gut microbiota with various chronic diseases such as obesity, CVD, diabetes, and cancer [34,35]. Further functional investigations are warranted to verify these potential mechanisms.

### Strengths and limitations of the study

In our study cohort, both whole grains and cereal fiber were correlated with high levels of physical activity and better health status, as well as with low BMI, low levels of smoking, and low intakes of alcohol and red meat. However, our results were less likely due to the potential confounding of these factors because careful adjustment for these factors in our analyses did not significantly change the results. Nevertheless, we acknowledge that the positive associations may still be related to residual confounding of non-measured covariates. Reverse causality might also affect the associations, since people with chronic disease might modify their eating habits by consuming healthy foods including those rich in whole grains and cereal fiber. In our analyses, however, we have excluded patients with cancer, heart disease, and diabetes at baseline and only analyzed the associations with incident cases. Whole grains and cereal fiber intakes were evaluated by self-report at a single time point. It is likely that dietary habits might change during the long (14 years on average) follow-up period, and such temporal patterns were not reflected in our analysis. Moreover, the observational nature of our study limits causality inference between intakes of whole grains or cereal fiber and mortality.

## Conclusions

Data from our study indicate that intake of whole grains and cereal fiber may reduce the risk of all-cause mortality and death from chronic diseases such as cancer, CVD, diabetes, respiratory disease, infections, and other causes. Disappearance or attenuation of whole grain associations with total mortality and death from chronic diseases after adjustment for cereal fiber intake suggests that cereal fiber partly accounts for the protective effects of whole grains on mortality.

## Abbreviations

CI: Confidence intervals
CVD: Cardiovascular diseases
FFQ: Food frequency questionnaire
HR: Hazard ratio
NIH: National Institutes of Health
