# Erratum: Consumption of whole grains and cereal fiber and total and cause-specific mortality: prospective analysis of 367,442 individuals

**DOI:** 10.1186/s12916-015-0338-z

**Published:** 2015-04-16

**Authors:** Tao Huang, Min Xu, Albert Lee, Susan Cho, Lu Qi

**Affiliations:** Department of Nutrition, Harvard School of Public Health, 665 Huntington Ave, Boston, 02115 MA USA; NutraSource (AWL), Royal Oak, 48073 MI USA; NutraSource (SSC), Clarksville, 21029 MD USA; Channing Laboratory, Department of Medicine, Brigham and Women’s Hospital and Harvard Medical School, 75 Francis St, Boston, 02115 MA USA

## Abstract

This is an Erratum to *BMC Medicine* 2015, **13**:59, highlighting previously undeclared competing interests and including more information in the acknowledgements section.

Please see related article: http://www.biomedcentral.com/1741-7015/13/59

## Erratum

### Authors’ corrected note

There was missing information in the competing interests and acknowledgements sections of our published article [1]. The erratum includes this information.

### Corrected text

The statement in the competing interests should read as follows: This study is funded by unrestricted research fund from NutraSource. Susan Cho is founder and owner of NutraSource and Albert Lee is an employee of NutraSource. The other authors declare that they have no competing interests.

The following information should be included in the acknowledgements section: The authors also thank Dr. Yi K Park at National Cancer Institute for her guidance in the study design.
